# Plasma and local expressions of CircRNA CDR1as are linked with disease severity in patients with non-traumatic osteonecrosis of femoral head

**DOI:** 10.1186/s13018-020-02129-z

**Published:** 2020-12-09

**Authors:** Bin Jiang, Shu-Hua Zhu, Ji-Yong Zeng, Zheng Mao

**Affiliations:** 1 Department of Orthopedics, Panyu Hospital of Chinese Medicine, Guangzhou, 511400 Guangdong Province China; 2 Department of Radiology, Guangzhou Panyu Central Hospital, Guangzhou, 511400 Guangdong Province China; 3 Department of Rehabilitation Medicine, The Third Affiliated Hospital, Southern Medical University, Zhongshan Road West No.183, Guangzhou, 510630 Guangdong Province China

**Keywords:** CircCDR1as, Non-traumatic osteonecrosis of femoral head, Disease severity

## Abstract

**Objective:**

To investigate the correlation of plasma and local expressions of Circ CDR1as with disease severity in patients with non-traumatic osteonecrosis of femoral head (ONFH).

**Methods:**

Ninety-nine non-traumatic ONFH patients receiving surgery and 99 healthy individuals were enrolled in our study. Plasma and local Circ CDR1as were detected using real-time quantitative PCR (RT-qPCR). Radiographic progression was determined using Association Research Circulation Osseous (ARCO) classification system. Harris hip score (HHS) and visual analogue scale (VAS) were used to assess the clinical severity. Receiver operating characteristic (ROC) curve was carried out to evaluate the diagnostic value of plasma Circ CDR1as with regard to the radiographic severity.

**Results:**

Plasma Circ CDR1as expressions were significantly higher in non-traumatic ONFH patients compared with healthy controls. In non-traumatic ONFH patients, there were no significant differences of CircCDR1as expressions between patients with alcohol-induced ONFH and steroid-induced ONFH. CircCDR1as in local necrotic tissue were significantly higher than adjacent non-affected tissue. Plasma and local Circ CDR1as expressions in patients with ARCO phase 4 were markedly upregulated compared with ARCO phase 3; plasma and local Circ CDR1as expressions in patients with ARCO phase 3 were markedly upregulated compared with ARCO phase 1/2. Plasma and local CircCDR1as expressions were positively associated with ARCO classification. In addition, plasma and local Circ CDR1as expressions were positively correlated with VAS and HHS scores. ROC curve analysis indicated that plasma Circ CDR1as may act as a decent marker for radiographic progression in non-traumatic ONFH patients.

**Conclusions:**

Both plasma and local expressions of CircRNA CDR1as are linked with disease severity in patients with non-traumatic ONFH.

## Introduction

Non-traumatic osteonecrosis of the femoral head (ONFH) is a common disease characterized by death of bone cells due to insufficient blood flow [1], leading to high disability rate and severe influence on life quality [2]. In the USA, 10,000–20,000 patients are newly found to be affected with the disease per year [3], and in China, 100,000–200,000 new cases were reported per year [4]. In China, patients with ONFH caused by alcoholism and glucocorticoids accounted for a large proportion of the patients. These patients often miss timely diagnosis and early treatment and undergo total hip arthroplasty (THA) in the late stage, with consequent multiple joint revisions and a huge medical cost [5–8].

Circular RNA (circRNA) is a new class of endogenously expressed non-coding RNA, which is characterized by covalently closed loop structures with neither 5' to 3' polarity nor polyadenylated tail [7]. They are common in mammalian cells and regulate gene expression at the transcriptional or post-transcriptional level by interacting with microRNAs (miRNAs) or other molecules [8–10]. Additionally, high abundance, relative stability, tissue/developmental stage-specific expression, and evolutionary conservation among species make circRNAs very attractive for clinical application [11]. Due to these characteristics, circRNAs have a great potential to serve as significant biomarkers to diagnose diseases. Recently, circulating circRNAs have been reported to be used as potential biomarkers for the diagnosis and prognosis of various diseases including cancer [12, 13], essential hypertension [14], diabetes [15], and amyotrophic lateral sclerosis [16], etc.

Derived from cerebellar degeneration-related protein 1 antisense transcript (CDR1AS), CDR1as is the best-known circRNA and has been uncovered as a regulator of various cellular processes [17]. Abnormal expression of CircCDR1as can be a cause of different kinds of diseases such as cancer [18], neurodegenerative [19] and autoimmune diseases [20], etc. Previous studies have shown that CircCDR1as plays important roles in the musculoskeletal system [21], and recent studies indicate that CircCDR1as may be involve in non-traumatic ONFH development. One previous study has demonstrated that Circ CDR1as could promote adipogenic and suppresses osteogenic differentiation of BMSCs in steroid-induced ONFH [22].

All these previous studies indicated that Circ CDR1as may act as a potential marker to evaluate non-traumatic ONFH progression. However, there is no report about circCDR1as as biomarker to explore the correlation of diagnosis and prognosis in non-traumatic ONFH. In the current study, we examined the expression level of circCDR1as in ONFH tissues and plasmas by real-time quantitative reverse transcription-polymerase chain reactions (qRT-PCRs). Then, tests were performed between clinical information and circRNA expression level by analysis of variance (ANOVA) or *t* test and correlation analysis, and a receiver operating characteristics (ROC) curve was established to estimate the value of circRNA expression as a biomarker in non-traumatic ONFH.

## Patients and methods

### Study subjects

A total of 99 (63 men, 36 women; age 50.2 ± 9.1) unrelated non-traumatic ONFH patients receiving hip preserving surgery or total hip replacement (THR) were enrolled at the Panyu Hospital of Chinese Medicine (Guangzhou, China) from July 2018 to July 2020. All the patients were diagnosed through symptoms, functional disability, and imaging findings. Meanwhile, 99 (65 men, 34 women; age 49.9 ± 9.7) unrelated healthy subjects receiving regular medical check were drifted as controls. Patients were diagnosed using anteroposterior and lateral pelvic radiographs and magnetic resonance images. GC-induced ONFH was defined by a history of a mean daily dose of ≥ 16.6 mg or a highest daily dose of 80 mg of predinosolone equivalent within 1 year before the development of symptoms or radiological diagnosis in asymptomatic cases. The inclusion of alcohol-associated ONFH patients included the following: patients should have a history of alcohol intake > 400 ml/week (320 g/week, any type of alcoholic beverage) of pure ethanol for more than 6 months; ONFH should be diagnosed within 1 year after alcohol intake of this dose; and patients should not have other risk factors. Patients with a demonstrable history of direct trauma or possible combined causes were excluded. Healthy control subjects were matched with patients for gender and age and enrolled from subjects attending routine medical checkups. This study was approved by our ethical committee. Informed consents were obtained from both groups.

### Tissue collection

For preserving surgery, marrow core decompression with bone grafting was used. Epidural anesthesia was used with the hip blocked up. A longitudinal incision was made on the external side of the femur 2 cm below the greater femur trochanter. Being monitored by the C-arm X-ray machine, the position of the guide pin was determined, and the guide pin was drilled into the center of the necrotic area beneath the femur head cartilage through the femoral neck from under the trochanter and screwed into the edge of lesion area below the head using a decompressor with tube core. The biopsy device was screwed into the lesion area, and the yellowish white wax-like loose diseased tissues (necrotic tissue) were taken out from the front end of the biopsy device and delivered for further study. For total hip arthroplasty, the femoral head was obtained according to the standard surgery procedure, and necrotic samples and adjacent non-necrotic samples were collected directly. The normal bone tissues were collected at least 3 cm away from the margin of necrotic tissue using the biopsy device.

### Sample collection and total RNA extraction

Serial blood specimens were collected from patients before and after treatment. All specimens were collected in Vacutainer EDTA tubes and centrifuged for 10 min at 1000×*g*, no later than 30 min after the collection of specimens for harvesting plasma under room temperature. All plasma samples were extracted immediately and were stored at − 80 °C for further analysis. Human bone samples (approximately 500 mg) were cryo-grinded under liquid nitrogen using a freezer-mill 6750 (SPEX Certiprep Inc. NJ, USA). 5 ml lysis/binding buffer from Dynabeads Oligo (dt)25 kit (Dynal Biotech ASA, Oslo, Norway) were added to the grinded bone tissue, and it was centrifuged 9000 rpm for 15 min at room temperature. These parated median layer containing nucleic acid was added to 1 ml oligo-dt coated paramagnetic particles from Dynabeads Oligo (dt)25 kit. Total RNA was extracted from each specimen using Trizol reagent (Invitrogen, Life Technologies Inc, Darmstadt, Germany) according to the manufacturer’s instruction. RNA concentration was determined by ScanDrop Nuclear Acid Analyzer (analytikjena, Germany). In order to verify the integrity of RNA, 3 μl total RNA of each sample was electrophoretically separated on a 1% denatured agarose gel and subsequently detected via Chemical Mp Imaging System (Bio-Rad, USA). If the peak area of 28S ribosome RNA (rRNA) was approximately twice than that of 18S rRNA, the integrity of total RNA was accepted and used for later experiments.

### Reverse transcription and quantitative polymerase chain reaction (qRT-PCR)

The relative expression levels of CircCDR1as were quantified by quantitative reverse transcription polymerase chain reaction (qRT-PCR). All primers used in this study were designed via primer 5.0 software (premier, Canada) and synthesized in Sangon Biotech (Shanghai, China) (Table 1). GAPDH was employed as the intrinsic control for CircCDR1as. qRT-PCR was performed in triplicate of each sample using Maxima SYBR Green qPCR Master Mix (Thermo Scientific, USA) on the CFX connect real-time system (Bio-Rad, USA). The primer sequence was listed below: CircCDR1as forward: 5’-TCAACTGGCTCAATATCCATGTC-3’, reverse: 5’-ACCTTGACACAGGTGCCAT-3’; GAPDH forward: 5’-ACTCCTCCACCTTTGACGC-3’; reverse: 5’-GCTGTAGCCAAATTCGTTGTC-3’. Relative expression level of CircCDR1as was calculated using the 2^− ΔΔCt^ method.

**Table 1:** Demographic data

	Non-traumatic ONFH patients (***n*** = 99)	Healthy controls (***n*** = 95)	***P*** value
Age (years)	51.1 ± 11.0	51.7 ± 10.7	0.244
Sex (F/M)	40/59	38/57	0.187
BMI (kg/m^2^)	23.7 ± 2.3	23.5 ± 1.5	0.246
Disease duration (months)	85.3 (3–250)	/	
VAS scores	5.0 ± 1.9	/	
HSS scores	67.6 ± 11.1	/	
ARCO stage (1 & 2/3/4)	34/35/30	/	
Plasma CircCDR1as expressions	1.93 ± 0.32	1.00 ± 0.16	< 0.001
Local CircCDR1as expression in necrotic vs non-necrotic tissue	2.20 ± 0.25 vs 1.00 ± 0.06	/	

All data are given as the mean value ± SD or median

### Definition of radiographic severity

All the patients were investigated by X-rays, bone scan, and magnetic resonance imaging (MRI) to ascertain the diagnosis and early diagnosis, and staging was used which includes radiographs, computed tomography (CT), bone scans, and MRI [23]. For MRI analysis, a GE Signa 1.5 T superconducting MR (USA) was used for the hip examination. The coronal T1-weighted images were selected to measure the necrotic area. The results were assessed by two blinded experienced radiologists. The kappa value was calculated for the consistency of the diagnosis. The result is favorable when the kappa value is ≥ 0.8. The detailed ACRO classification was listed below: stage 0, diagnostic imaging results are normal; stage 1, conventional radiography and computed tomography results are normal, but MRI, scintigraphy, or both indicate the presence of osteonecrosis; stage 2, radiography demonstrates regions of irregularity, such as mottling, osteolysis, and sclerosis, but the femoral head is spherical on the anterior-posterior and lateral views; stage 3, the “crescent sign,” a fine radiolucent subchondral fracture line, is apparent on radiographs, and the femoral head mechanically fails; and stage 4, flattening of the femoral articular surface, narrowing of the joint space, and changes in the acetabulum are evident.

### Definition of clinical severity

Pro-operative clinical assessments were conducted using the visual analogue scale (VAS) [24] score and Harris hip score (HHS) [25]. For VAS score, a horizontal line of 10 cm is drawn on the of the paper [24]. At the beginning of the horizontal line is 0, indicating no pain. The end of the line is 10, suggesting extremely pain. The middle part shows different degrees of pain. The patient was asked to mark the horizontal line according to the patient’s self feeling to indicate the degree of pain. The HHS is an outcome tool typically used to evaluate hip function following total hip replacements [25]. The HHS comprises 10 items grouped into four domains: pain (1 item, 0–44 points); function (7 items, 0–47 points); absence of deformity (1 item, 4 points); and range of motion (2 items, 5 points). The score has a maximum of 100 points, and higher scores indicate less dysfunction and better outcomes. A total score of < 70 was considered as poor, 70–80 as fair, 80–90 as good, and 90–100 as an excellent result.

### Statistical analysis

All obtained data were analyzed using the Graphpad Prism 8.0 software (San Diego, CA, USA). The Kolmogorov-Smirnov test was operated for testing the distribution (normal or non-normal) of the variables. Normal distribution was expressed as mean ± standard deviation, whereas non-normally distributed continuous variables were expressed as median (95% CI). We determined the differences of CircCDR1as levels between the two groups using student’s *t* test or Mann–Whitney test, whereas one-way ANOVA or Kruskal–Wallis test were used for three or more groups followed by Tukey or Tamhan’s test for post hoc analysis. The Pearson rank or Spearman correlation test was employed to examine the relationship between plasma CircCDR1as expressions and ARCO classification as well as VAS score and HSS score. ROC curve analysis was employed to determine the diagnostic value of CircCDR1as with regard to ARCO classification. *P* values less than 0.05 were regarded as significant.

## Results

### Basic clinical statistics

The non-traumatic ONFH patients included 59 males and 40 females with their ages ranged from 20 to 70 years. The disease course ranged from 3 to 250 months. The main symptoms were all unilateral or bilateral hip pain and functional disability. There were no significant differences of age, sex distribution, and BMI between non-traumatic ONFH patients and healthy controls (Table 1).

### Plasma CircCDR1as expressions in non-traumatic ONFH patients

**Fig. 1:**
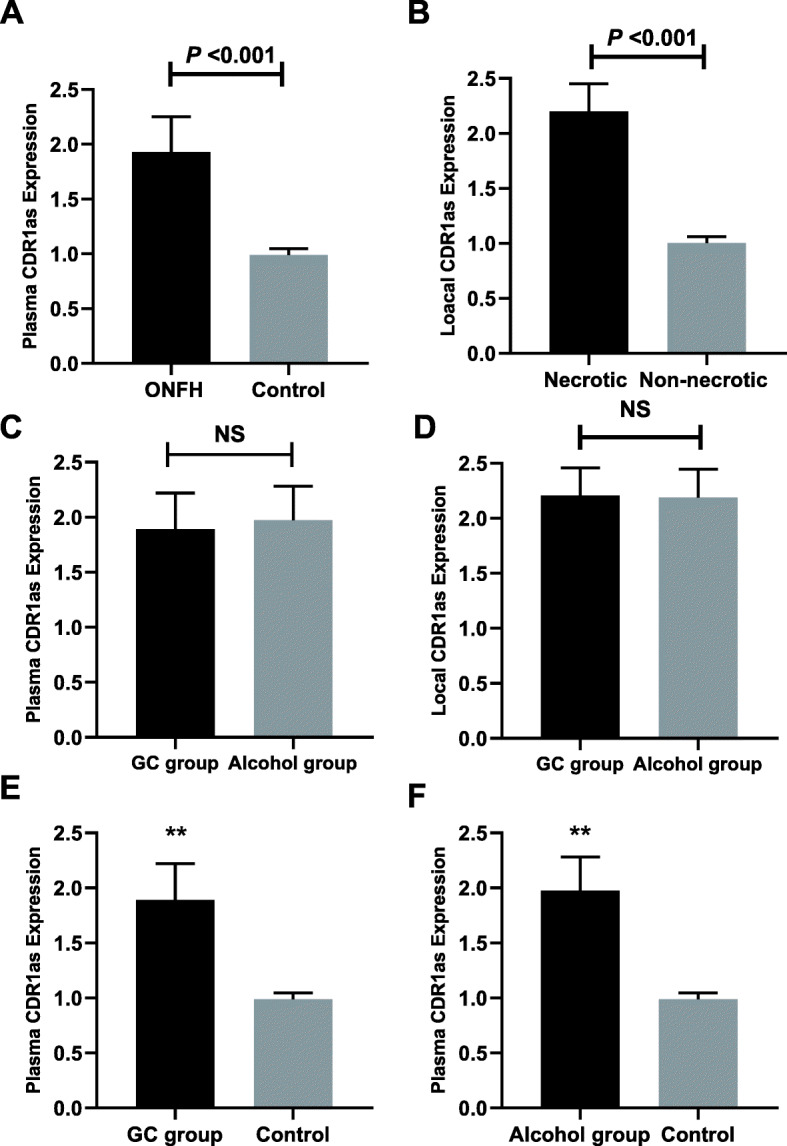
**a** Comparison of plasma CDR1as expressions between non-traumatic ONFH and control. **b** Comparison of local CDR1as expressions between necrotic tissue and non-necrotic tissue in non-traumatic ONFH patients. **c** Comparison of plasma CDR1as expressions between GC group and alcohol group. **d** Comparison of local CDR1as expressions between GC group and alcohol group. **e** Comparison of plasma CDR1as expressions between GC group and control. **f** Comparison of plasma CDR1as expressions between alcohol group and control

As depicted in Fig. 1, non-traumatic ONFH patients demonstrated significantly upregulated plasma CircCDR1as expressions compared with healthy controls (1.93 ± 0.32 vs 1.0 0 ± 0.06, *P* < 0.001) (Fig. 1a). According to the etiological factors of non-traumatic ONFH, patients were attributed into steroid-induced (52 cases) and alcohol-induced (47 cases) conditions. There was no significant difference in plasma and local CircCDR1as expressions between GC-induced and alcohol-induced ONFH patients (plasma: 1.89 ± 0.33 vs 1.97 ± 0.31, *P* = 0.199; local: 2.20 ± 0.25 vs 2.19 ± 0.27, *P* = 0.710) (Fig. 1b). In non-traumatic ONFH patients, we found local CDR1as expressions were 3.56-fold higher than paired plasma samples (3.56 ± 0.12 vs 1.00 ± 0.05, *P* < 0.001). Moreover, CircCDR1as expressions in necrotic tissues were significantly higher than adjacent non-necrotic tissue (2.20 ± 0.25 vs 1.00 ± 0.06, *P* < 0.001) (Fig. 1d). Plasma CircCDR1as expressions in both alcohol group and steroid group were significantly higher than those in the control group (GC group vs control: 1.89 ± 0.33 vs 1.00 ± 0.06, *P* < 0.001 ; alcohol group vs control: 1.97 ± 0.31 vs 1.00 ± 0.06, *P* < 0.001) (Fig. 1e, f).

### Plasma and local CircCDR1as expressions with ARCO classification

**Fig. 2:**
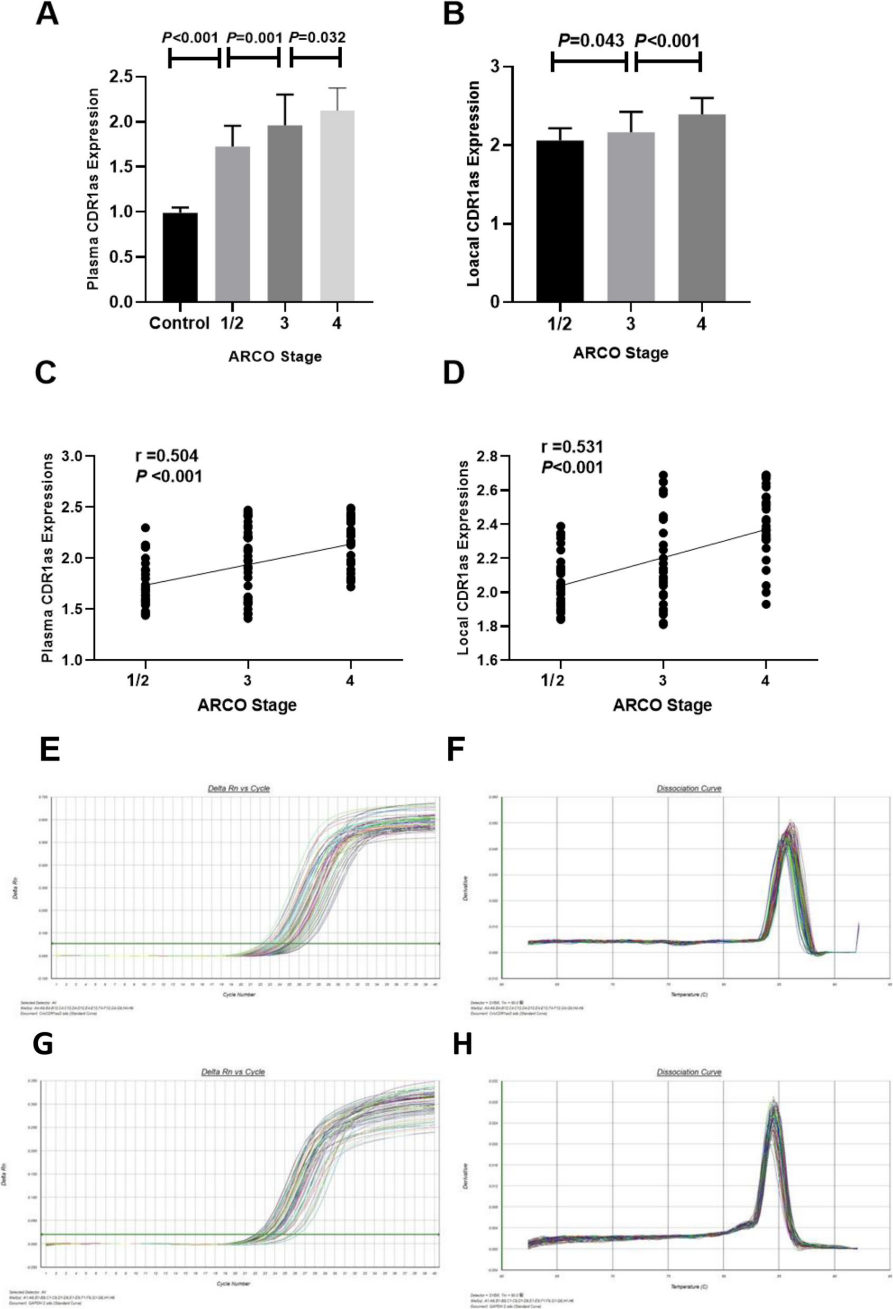
**a** Comparison of plasma CDR1as expressions among different ARCO stages and controls. **b** Comparison of local CDR1as expressions among different ARCO stages. **c** Correlation of plasma CDR1as expressions with different ACRO stages. **d** Correlation of local CDR1as expressions with different ACRO stages. **e**, **f** Representative strips of CDR1as detected by RT-PCR in plasma between two groups. **g**, **h** Representative strips of CDR1as detected by RT-PCR in local tissue between two groups

Plasma and local CircCDR1as expressions in 99 non-traumatic ONFH patients with different ARCO stage are demonstrated in Fig. 2.Non-traumatic ONFH patients were classified into three groups on the basis of the ARCO stage. The non-traumatic ONFH patients included 34 patients with ARCO stage 1/2, 35 with stage 3, and 30 with stage 4. Non-traumatic ONFH patients with ARCO stage 4 had significantly higher plasma CircCDR1as expressions than ARCO stage 3 (2.13 ± 0.25 vs 1.96 ± 0.34, *P* = 0.032) (Fig. 2a). In addition, non-traumatic ONFH patients with ARCO stage 3 demonstrated markedly increased plasma CircCDR1as expressions compared with ARCO stage 1/2 (1.96 ± 0.34 vs 1.73 ± 0.23, *P* = 0.001) (Fig. 2a). We also found that plasma CircCDR1as expression was significantly higher than control (1.73 ± 0.23 vs 1.00 ± 0.06) (Fig. 2a). Plasma CircCDR1as expressions were positively associated ARCO stage (*r* = 0.504, *P* < 0.001) (Fig. 2c).

Besides, we also found non-traumatic ONFH patients with ARCO stage 4 had drastically higher local CircCDR1as expressions than those with ARCO stage 3 (2.39 ± 0.21 vs 2.17 ± 0.26, *P* < 0.001) (Fig. 2b). Moreover, non-traumatic ONFH patients with ARCO stage 3 demonstrated markedly higher local CircCDR1as expressions compared with ARCO stage 1/2 (2.17 ± 0.26 vs 2.06 ± 0.16, *P* = 0.043) (Fig. 2a). Local CircCDR1as expressions were also positively related to ARCO stage (*r* = 0.531, *P* < 0.001) (Fig. 2d).

### Plasma and local CircCDR1as expressions with symptomatic severity

**Fig. 3:**
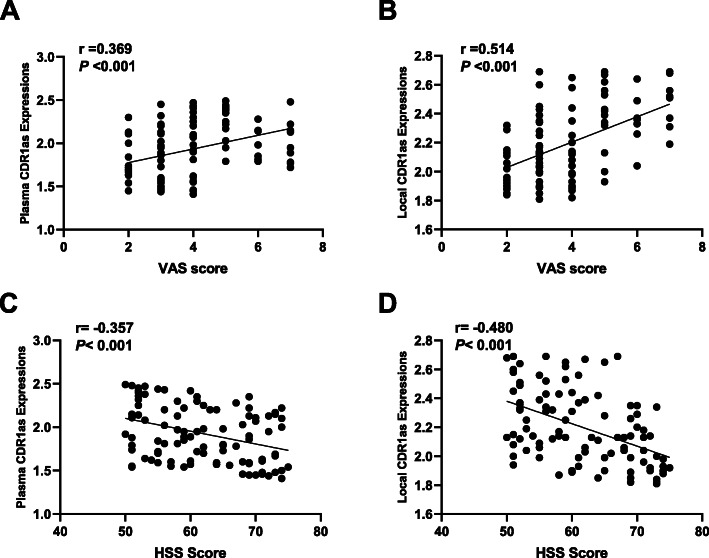
**a** Correlation of plasma CircCDR1as expressions with VAS score. **b** Correlation of local CircCDR1as expressions with VAS score. **c** Correlation of plasma CircCDR1as expressions with HSS score. **d** Correlation of local CircCDR1as expressions with HSS score

Next, we investigated the correlation of plasma and local CircCDR1as expressions with clinical severity determined by VAS and HSS scores. On the one hand, we found plasma and local CircCDR1as expressions were positively associated with VAS scores (plasma: *r* = 0.369, *P* < 0.001; local: *r* = 0.514, *P* < 0.001) (Fig. 3a, c). On the other hand, plasma and local CircCDR1as expressions were significantly and negatively related to HSS scores (plasma *r* = − 0.357, *P* < 0.001; local *r* = − 0.480, *P* < 0.001) (Fig. 3b, d).These findings implicated that non-traumatic ONFH patients with higher plasma and local CircCDR1as expressions might have more pain and worse functions.

### ROC curve analysis

**Fig. 4:**
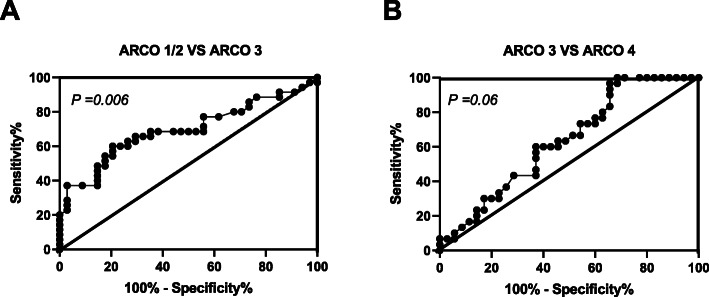
**a** ROC curve analysis of ARCO stage 1/2 vs 3 with regard to CircCDR1as expressions. **b** ROC curve analysis of ARCO stage 3 vs 4 with regard to CircCDR1as expressions

We last performed ROC curve analysis to detect the diagnostic value of whether plasma CircCDR1as may play as a potential diagnostic biomarker for ARCO stage in non-traumatic ONFH patients. As demonstrated in Fig. 4, increased PACAP showed significant AUC for ARCO stage 1/2 vs stage 3 (AUC = 0.695, *P* = 0.006) (Fig. 4a), but non-significant AUC for ARCO stage 3 vs stage 4 (AUC = 0.635, *P* = 0.06) (Fig. 4b). These findings implicated that increased plasma CircCDR1as might serve as an early to medium instead of late diagnostic marker to evaluate disease development of non-traumatic ONFH.

## Discussion

Our present study explored the correlation of plasma and local CircCDR1as expressions with disease severity of non-traumatic ONFH. We demonstrated for the first time that plasma CircCDR1as expressions were significantly higher compared with healthy controls. Meanwhile, in non-traumatic ONFH patients, local CircCDR1as expressions in necrotic tissue were markedly increased than in adjacent non-necrotic tissue. Moreover, increased plasma and local CircCDR1as expressions were significantly and positively correlated with ACRO stage. In addition, we also found both plasma and local CircCDR1as expression were positively related to VAS score and negatively correlated with HHS score. ROC curve analysis showed that increased plasma CircCDR1as expression may act as an early biomarker for ACRO stage. To our best of knowledge, this is the first time we identified circular RNA as a biomarker in the progression of non-traumatic ONFH.

The typical pathological features of non-traumatic ONFH include decreased density of the femoral head, cystic degeneration of the bone, fracture of the trabecular structure and deformity and collapse of the femoral head [6]. However, bone injuries that may not be detected by radiographic examinations are often present during the initial stage of non-traumatic ONFH. Therefore, to seek potential biomarkers as early detective method may serve as an alternative way in diagnosis of non-traumatic ONFH. Majority of CircRNAs are evolutionarily conserved across species and are stable both in serum and in tissue .Therefore, CircRNAs could be used as potential biomarkers to reflect disease severity and predict prognosis.

Previous studies have shown that CircRNAs have many important biological functions. First, CircRNAs act as sponges of miRNAs to regulate the function of miRNA target genes [26, 27]. Second, they directly regulate the level of other RNAs through complementary base-pairing [28]. Third, CircRNAs can regulate the activity of proteins by binding to them directly [29]. Finally, a small number of CircRNAs can also be translated into a template to guide protein synthesis [30, 31]. Therefore, CircRNAs play important regulatory roles in organisms by regulating the expression of different genes, and how this function relates to the progression of non-traumatic ONFH has attracted extensive attention.

Recent studies implicated CircRNAs may have an important role in ONFH, which is closely, to their functions, related to the activity of osteoblasts and osteoclasts, endothelial cell damage, weakened osteogenic differentiation, and increased adipogenic differentiation of BMSCs [32]. Therefore, CircRNAs may have a role as diagnostic biomarkers in ONFH. For example, CircRNA 0003575 is upregulated in oxidized low-density lipoprotein-induced HUVECs, and promotes HUVEC proliferation and angiogenesis [33]. CircRNA 0010729 mediates vascular endothelial cell apoptosis and proliferation by targeting the miR-186/hypoxia inducible factor-1α axis [34]. Kuang et al. [35] showed that CircRNA ubiquitin-specific protease 45 can enhance the PTEN expression through sponging miR-127-5p, which inhibits the protein kinase B pathway and regulates bone mass in rat steroid-induced ONFH. These findings indicate CircRNA may function as a miRNA sponge to contribute to pathophysiology of ONFH.

In the present study, we assessed the potential expressions of plasma and local CDR1as, a CircRNA with the disease severity of non-traumatic ONFH. First, we found plasma and local CDR1as was significantly increased in non-traumatic ONFH patients. One previous study found that CircRNA CDR1as was overexpressed in SONFH-BMSCs with decreased osteogenic differentiation [22]. Furthermore, the expression of CircCDR1as was negatively correlated with the osteogenic differentiation process of BMSCs (bone marrow mesenchymal stem cells) [22]. Bioinformatics results indicated that CircCDR1as could promote adipogenic differentiation while attenuating osteogenic differentiation of BMSCs [22], indicating CircCDR1as may play important roles in the progression of non-traumatic ONFH. We also found there were no differences of CircCDR1as expressions between steroid group and alcoholic group, implicating the change of CircCDR1as expressions in non-traumatic ONFH regardless of the etiology. We next found that both increased local and plasma CircCDR1as expressions were correlated with more pain as well as worse function. Cai found CircCDR1as could target miR-135a-5p to neuropathic pain in CCI rats via inflammation and autophagy [36].

There were several limitations that should be noted. Firstly, this study was carried out and single-centered study with relatively sample size in China, further multicenter studies with more samples are needed to identify the results. Secondly, since this is a cross-sectional study, the causal relationship between increased expression CircCDR1as and development of ONFH could not be illuminated. Therefore, the detailed mechanisms that unveiling CircCDR1as in ONFH should be explored in further study. Thirdly, we did not measure CircCDR1as expressions in healthy individual’s femoral head due to ethical reasons. Hence, we were not able to compare the rate and amount of increment between plasma CDR1 and local CDR1 to show whether plasma CDR1 or local CDR1 is a more useful determinant in that the we used different controls. Last, we did not explore the potential mechanism of CircCDR1as in the development of non-traumatic ONFH.

## Conclusion

In conclusion, we found higher levels of plasma CircCDR1as in non-traumatic ONFH patients compared with healthy controls. Also, plasma and local CircCDR1as expressions were positively correlated with radiological and symptomatic severity. Further studies investigating the intensive mechanisms of CircCDR1as in non-traumatic ONFH would provide a better insight about the potential clinical target of CircCDR1as.Therapies that targeting CircCDR1as and its related regulating signaling ways may serve as a potential interventional method in non-traumatic ONFH.

## Data Availability

The datasets used and/or analyzed during the current study are available from the corresponding author on reasonable request.

## Abbreviations

ONFH: Osteonecrosis of femoral head
ACRO: Association Research Circulation Osseous
BMSC: Bone marrow mesenchymal stem cells
THA: Total hip arthoplasty
CDR1AS: Cerebellar degeneration-related protein 1 antisense transcript
ROC: Receiver operating characteristic
AUC: Area under curve
VAS: Visual analogue scale
HHS: Harris hip score
